# Management of Severe Pediatric Lower Lip Defect After Canine Bite with Polyhexamethylene Biguanide (PHMB), Full-Thickness Skin Graft (FTSG) and Compression Foam: A Case Report

**DOI:** 10.3390/children12101308

**Published:** 2025-09-28

**Authors:** Aba Lőrincz, Hermann Nudelman, Anna Gabriella Lamberti, Attila Vástyán, Enikő Molnár, Gábor Pavlovics, Gergő Józsa

**Affiliations:** 1 Division of Pediatric Surgery, Traumatology, Urology and Pediatric Otolaryngology, Department of Pediatrics, Clinical Complex, University of Pécs, 7 József Attila Street, 7623 Pécs, Hungary; aba.lorincz@gmail.com (A.L.); nuhwaao.pte@tr.pte.hu (H.N.); lamberti.anna@pte.hu (A.G.L.); vastyan.attila@pte.hu (A.V.); molnar.eniko@pte.hu (E.M.); 2 Department of Thermophysiology, Institute for Translational Medicine, Medical School, University of Pécs, 12 Szigeti Street, 7624 Pécs, Hungary; 3 Plastic and Burn Surgery Department, Surgical Clinic, Clinical Complex, University of Pécs, Ifjúság Street 13, 7624 Pécs, Hungary; pavlovics.gabor@pte.hu

**Keywords:** pediatric, lip, dog, bite, polyhexamethylene biguanide, polyhexanide, PHMB, graft, FTSG

## Abstract

**Highlights:**

**What are the main findings?**

- Primary closure was achieved in a large, lower-lip defect after a dog bite using an integrated approach (topical polyhexanide, FTSG secured with a polyurethane compression foam, oral antibiotics).
- Function remained intact; at 12 months, modest esthetic sequelae persisted (dyschromia, shallow border indentation, vellus hairs).

**What are the implications of the main findings?**

- This pediatric case shows that the combined intervention can yield stable function with moderate esthetic compromise in a contaminated, extensive lower-lip injury.
- Component-level effects are undetermined; comparative studies with validated outcomes are needed.

**Abstract:**

**Introduction**: Pediatric lower-lip dog bite injuries are challenging due to contamination, tissue loss, and the need to maintain function, appearance, and psychological well-being. This single case describes immediate definitive closure using sharp debridement with adjunct polyhexanide (PHMB), a full-thickness skin graft (FTSG), and a polyurethane (PU) compression foam bolster. **Methods**: A 10-year-old boy with a severe contaminated lower-lip defect underwent debridement and 0.04% PHMB irrigation. An upper-arm FTSG was inset and compressed with a suture-anchored PU dressing. Topical PHMB gel was used perioperatively and for seven days after bolster removal. Oral antibiotics were given for five days. The patient was discharged eight days after the injury with detailed wound care instructions. **Results**: Immediate definitive closure was achieved with complete graft survival and no infection, necrosis, unplanned early dressing changes, or reoperations. At 12 months, oral competence, speech, lip mobility, and contour were preserved. However, mild residual esthetic differences remained (dyschromia, shallow border indentation, vellus hairs on the graft). **Conclusion**: In this single descriptive case, primary closure of a lower-lip injury with the combined intervention was associated with an uncomplicated functional course and manageable esthetic trade-offs at 12 months. These observations are descriptive; comparative studies with standardized functional, esthetic, and psychosocial measures are needed.

## 1. Introduction

Canine attacks are a common source of trauma in pediatric patients, with a higher than 50% lifetime prevalence [1]. Facial injuries account for approximately 60% of all dog bites (i.e., vulnus morsum canis), mainly in toddlers, while a distinct trend toward increased risk for upper extremity injuries in older children has been observed [2,3,4]. Among head traumas, the lower lip (9–46% of all cases) is most frequently affected due to its protrusion and vulnerability during interactions with animals. The cheek (~26%) and the nose (10.3–24.8%) follow [5]. The lower lip, in particular, is prone to deep, complex injuries that may involve the skin, mucous membranes, and the underlying muscles or even bones. If fractures are present, they most frequently affect the nasal bone (29%). Associated injuries include eyelid (41%), canalicular (18%), and facial-nerve trauma (24%). [6]. Recent studies suggest that facial dog bites are most prevalent throughout the summer in male children aged 10 or younger [1]. In modern times, canine attacks almost equally arise from provoked aggression or play, generally (~90%) prompted by a familiar animal [7].

Current protocols for facial dog bite injuries prioritize early decontamination, debridement, and reconstruction [8,9,10]. Given the polymicrobial nature of canine bites, prophylactic antibiotics and vigilant post-surgical monitoring are recommended, particularly when the animal’s vaccination status is unknown. Rabies (inflicted by Lyssavirus) was first noted by ancient Sumerians and linked to fatal infections after rabid animal saliva contact. Although it remains a concern in under-vaccinated regions, most bites do not transmit the disease. Nevertheless, zoonotic risks continue to be critical considerations in treatment [11].

Polyhexamethylene biguanide (polyhexanide or PHMB) is a cationic polymer that uniquely targets a wide range of pathogens by binding to their negatively charged membranes, destabilizing them, and causing leakage of essential intracellular components [12]. In wound care—especially for dog bite injuries with their complex, polymicrobial contamination—PHMB serves both as an irrigating solution and as a gel [13]. Its low cytotoxicity preserves host tissue integrity, which is critical for successful graft adherence, and its prolonged antimicrobial action reduces the need for frequent dressing changes [14]. Our department routinely utilizes PHMB for burn management [15,16], while other institutions have reported favorable outcomes when administered on heel lacerations [17], pin sites [18], or during negative-pressure wound therapy (NPWT) instillation [19]. However, PHMB activity can be reduced by high organic burden, and prolonged use carries a modest risk of local irritation or allergic reaction. Irreversible ocular damage may also occur if applied directly (conjunctivitis, epithelial defect, or even progressive cataract) [20].

Selecting an optimal reconstructive technique is significantly complicated by the unique healing dynamics of pediatric skin [21]. Although children exhibit superior wound healing and scar remodeling compared to adults, they remain vulnerable to unpredictable tissue contraction and hypertrophic scarring [22]. Moreover, the highly vascular perioral region, while enhancing healing, can exacerbate bleeding if not carefully managed, thereby increasing the risk of infection and other complications. Although small, superficial wounds are often amenable to primary closure, larger defects involving composite tissue loss demand advanced reconstructive techniques [10,23,24]. Suturing is tailored to the wound’s depth and extent of tissue damage. Immediate closure of facial dog bite wounds is generally recommended only when there is no infection [8,9]. Full-thickness skin grafting (FTSG) remains a well-established method for managing extensive defects, while severe injuries affecting muscles or bones may often necessitate the use of composite tissue flaps [5].

Beyond physical healing, the psychological impact of facial trauma on children can be profound. Visible scars may contribute to self-esteem issues, social withdrawal, and long-term emotional distress, increasing the risk of anxiety and depression [25,26]. This reality underscores the necessity of an integrated, multidisciplinary approach that brings together pediatric surgeons, speech therapists, and psychologists to address both anatomical restoration and psychosocial well-being.

Despite advances in wound care, optimal reconstructive approaches for pediatric lower lip trauma after a canine attack remain disputed. Although there are attempts at protocolizing it, it has not yet become widespread [23]. Our report describes a novel method that employs PHMB both as an irrigant and as a sustained-release gel, applied beneath a polyurethane (PU) compression foam dressing following FTSG for a lower lip dog bite. Dual application of PHMB leverages its low cytotoxicity and prolonged antimicrobial action to control infection and enhance graft adherence, possibly improving wound bed integrity and clinical outcomes. Therefore, this study intends to stimulate further research into the role of PHMB in pediatric facial reconstruction.

## 2. Materials and Methods

### 2.1. The Study Design and Patient Selection

Between July 2024 and January 2025, the Division of Pediatric Surgery, Traumatology, Urology, and Pediatric Otolaryngology, Department of Pediatrics at the University of Pécs, Clinical Complex was involved in an ongoing case report analyzing the treatment of a pediatric lower lip dog bite injury employing dual-phase PHMB irrigation and FTSG with a PU compression foam and assessing its postoperative complications. Written informed consent was acquired from the guardian of the patient to publish the results.

### 2.2. Operative Algorithm

Following pain management with 50 mg oral diclofenac (Cataflam^®^, Novartis AG, Basel, Switzerland), midazolam sedation (Dormicum^™^, Egis Gyógyszergyár Zrt, Budapest, Hungary), and 600 mg amoxicillin-clavulanate antibiotic injection (Augmentin^™^, GlaxoSmithKline, London, UK), standard preoperative preparation ensued. General anesthesia was introduced through a laryngeal mask. Then, the injury was cleaned with sterile saline and irrigated with a 0.04% PHMB solution (LAVANID^®^—2, SERAG-WIESSNER, Naila, Germany). Next, wound debridement was performed to remove devitalized tissue. FTSG was harvested from the left upper arm and transplanted onto the affected area to reconstruct the defect, in conjunction with a fascial repair to optimize structural integrity. Absorbable sutures (4/0 Radic Fast^™^, SC Medical Technology Kft, Pécs, Hungary) secured the graft. Then, it received sterile preserved 0.04% PHMB gel (LAVANID^®^—Wound Gel, SERAG-WIESSNER, Naila, Germany) and was stabilized with an external, open-pore, hydrophobic PU compression foam dressing (Small Foam Dressing Kit (100 × 80 × 30 mm), WoundRX Medical LLC, Southbury, CT, USA), which is mainly used for NPWT. After primary donor site closure, postoperative immobilization was maintained with the sponge dressing.

Postoperatively, antibiotic prophylaxis was continued three times daily for five days, and analgesia for three days. One week after surgery, the foam dressing and fixation sutures were removed under total intravenous anesthesia (TIVA), followed by PHMB gel application for a week. On the eighth day, the patient was discharged with detailed postoperative care instructions, including topical wound management with petrolatum-based ointment for at least 3–4 weeks to prevent dehydration of the rubor labii. Additionally, an extractum cepae, allantoin, and heparin-containing gel (Contractubex^®^, Merz Therapeutics GmbH, Frankfurt am Main, Germany) was applied for 11 months to minimize scarring. Scar massage therapy was added to the regimen four months after surgery to improve soft tissue elasticity and minimize any potential contracture. Given the extent of the initial injury, long-term follow-up must be planned to assess healing outcomes and evaluate the potential need for secondary reconstructive procedures.

## 3. Results

A 10-year-old boy presented with a large, contaminated lower-lip defect after a bite from a known, unvaccinated dog. He was hemodynamically stable without systemic infection; routine preoperative labs were normal. The dog remained well during the 10-day observation, so no rabies post-exposure prophylaxis was given. Past history (cetirizine use, prior tonsillectomy, inguinal hernia repair, and tooth extraction) did not alter perioperative management.

**Figure 1:**
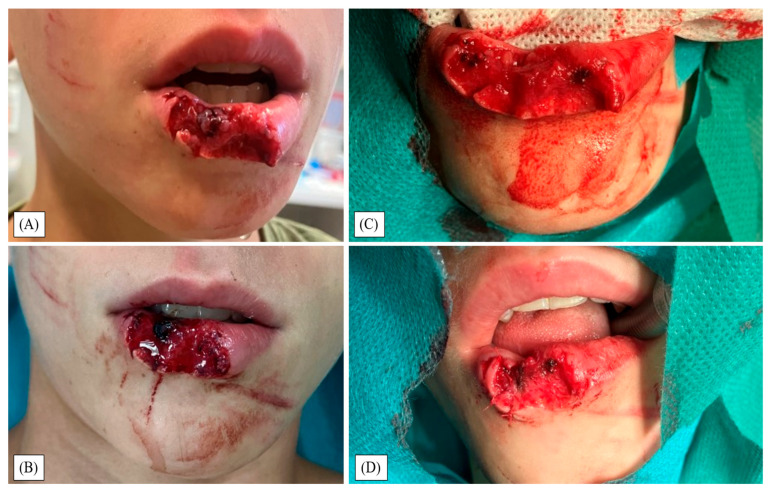
(**A**) First hospital examination showing a right-sided labium inferius avulsive laceration extending below the vermilion; measured 30 × 15 × 6 mm. (**B**) Preoperative view in the operating room. (**C**) After sharp debridement and lavage using 0.04% polyhexanide (PHMB) solution. (**D**) Defect reduction and edge regularization with approximation sutures.

Approximately half of the lower lip was involved, extending across the vermilion into the cutaneous lip; the defect measured 30 × 15 × 6 mm (Figure 1A–C). Due to size and contour considerations, immediate definitive closure at the initial surgery was planned using a combined approach.

**Figure 2:**
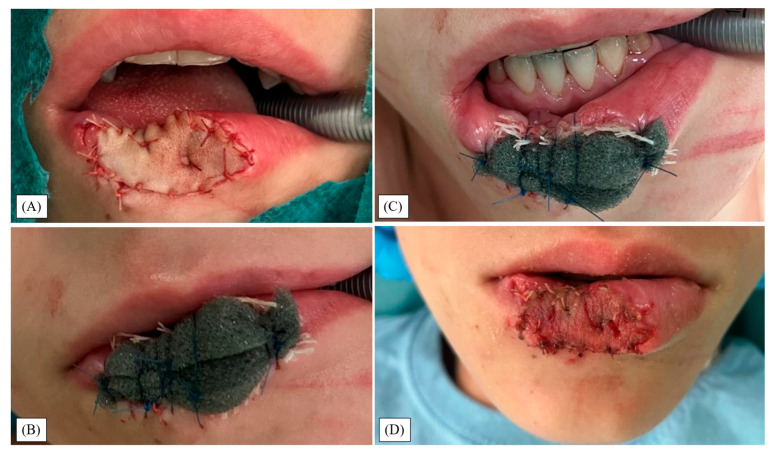
(**A**) Full-thickness skin graft (FTSG) harvested from the left upper arm was inset over fascia/orbicularis and secured with absorbable sutures. (**B**,**C**) Suture-anchored polyurethane (PU) compression dressing, demonstrated with the mouth closed (**B**) and open (**C**). PHMB gel was applied over the graft before dressing placement. (**D**) Postoperative day 7: dressing removal revealed a viable, adherent graft.

Under general anesthesia using a laryngeal mask and antibiotic prophylaxis, debridement was performed combined with PHMB solution irrigation (Figure 1B,C). Wound margins and free wound flaps were brought closer together with sutures to create a smaller injury with sharper edges (Figure 1D). Next, the defect was reconstructed with an FTSG harvested from the left upper arm and absorbable sutures (Figure 2A).

Subsequently, the PHMB gel was smeared on the graft surface, and it was externally compressed with a suture-anchored foam dressing (Figure 2B,C). Postoperatively, the patient received antibiotics for five days and pain management for three days. One week after surgery, the graft showed no signs of infection and displayed successful integration upon suture and dressing removal (Figure 2D). PHMB gel application continued for a week after detaching the compression foam. He was discharged on the eighth day, in good general condition, with specific topical wound management directives.

**Figure 3:**
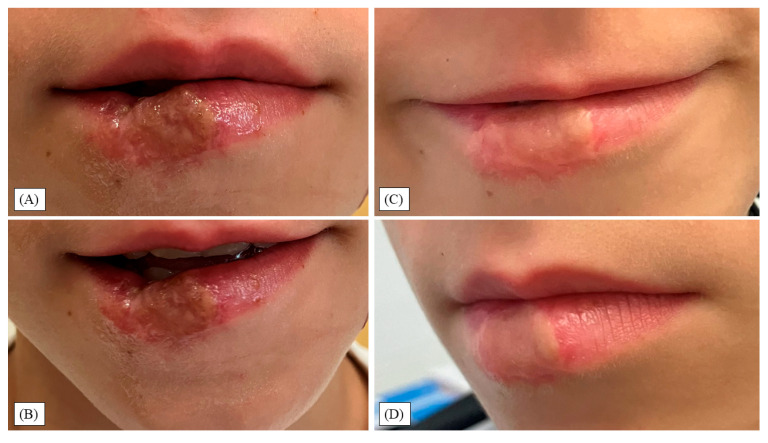
(**A**,**B**) Two-week review: grafted lower lip with mild perilesional erythema, edema, and adherent yellow-tan fibrin/pseudomembrane compatible with early epithelialization. (**C**,**D**) Four-month follow-up: symmetric lower-lip motion with normal speech and minimal scar thickening; scar massage and physical therapy (PT) commenced at this stage.

At the two-week outpatient review, the FTSG was fully adherent and covered by a thin fibrinous/pseudomembranous layer consistent with early epithelialization (Figure 3A,B). Mild marginal erythema and edema were present without purulent drainage, fluctuance, malodor, or tissue necrosis.

At four months, the site was fully epithelialized with preserved oral competence, speech, lip mobility, and contour (Figure 3C,D). Mild soft-tissue firmness was palpable, without hypertrophic scarring or contracture, consistent with normal postoperative healing. Scar massage and physical therapy (PT) were initiated at this visit.

**Figure 4:**
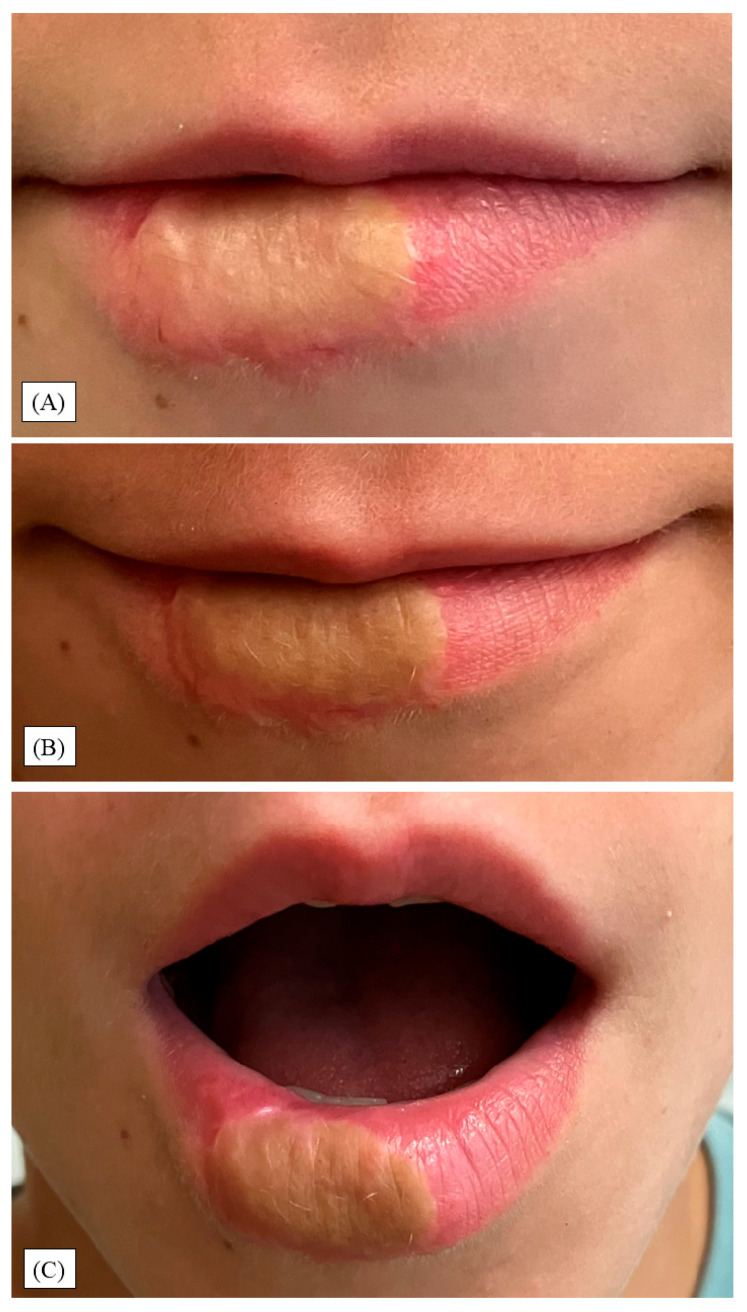
(**A**) Eight-month control: stable graft with a minor linear indentation at the graft–vermilion junction and mild color mismatch. (**B**,**C**) Twelve-month review: slightly softened contour irregularity, more apparent dyschromia, and fine vellus hairs within the graft; patient and parent satisfaction remained high.

At the eight-month follow-up appointment, the child presented with stable graft morphology and mostly maintained anatomical contours (Figure 4A). Functional parameters remained optimal: speech articulation was unaffected and lip motion and smiling dynamics were symmetrical, without compensatory muscle behavior. Although the previously mild firmness was reduced to being minimal, the graft displayed a slightly accentuated color contrast relative to the vermilion zone. Also, a small, narrow, linear indentation was detectable, originating from the point of injury, which was closest to the oral mucosa.

During the one-year control visit, the indentation and lip contour marginally improved (Figure 4B,C). However, a few fine vellus hairs appeared at the transplant site, and the discoloration became more visible. Importantly, these features did not result in discomfort, hypersensitivity, or mechanical restriction. Additionally, there were no signs of infection, ulceration, or contracture formation. Parental and patient satisfaction remained high. No clinical or behavioral indicators of psychosocial distress were observed, and the child reintegrated fully into school and social activities. Although no standardized quality-of-life metrics were recorded, both verbal and non-verbal assessments by caregivers and clinical staff indicated favorable psychosocial adaptation.

## 4. Discussion

Reconstructing a complex defect of the lower lip requires a careful balance between maintaining oral competence and restoring anatomical integrity [8,10,24]. Our choice of FTSG with external compression reflected case-specific priorities. In a large, contaminated, avulsive defect in a small mouth, maintaining the oral aperture, lip mobility, and border geometry at a single sitting was paramount. An immobilized graft permitted resurfacing without adding bulk and with predictable postoperative care, while avoiding traction across the commissures during early healing. This is a clinical rationale, not a claim of superiority; we did not compare FTSG against mucosal grafts or flaps and cannot determine relative performance. Multiple mucosal and cutaneous flap options remain suitable depending on defect geometry, vascular bed, and commissure involvement, and technique selection should be individualized in children with consideration of growth.

When appropriately immobilized, FTSG can offer reliable vascular integration. It is well-established for providing durable coverage with minimal donor site morbidity in other sites, though it may result in contour irregularities, pigmentary mismatch, and secondary contraction in growing children [27,28]. At eight months, graft color approximated the perioral skin; however, partial effacement of the lower vermilion border was evident. Pigmentary variation appeared more pronounced at 12 months, possibly reflecting cumulative sun exposure. Border irregularities were attributable to minor scarring; these softened with massage and PT. Fascial repair in combination with skin grafting may have contributed to structural reinforcement and helped limit the postoperative distortion of the lip contour. Vellus hairs were apparent on the grafted vermilion at the one-year follow-up and may become more conspicuous after puberty if they terminalize. In our patient, early outcomes favored continued conservative management; no additional surgical procedures were indicated, though elective esthetic refinements (laser hair removal, mucosa transplant) remain available if priorities evolve.

Vermilion correction on smaller defects is best achieved with mucosal grafts and flaps, which are considered first-line treatments. Buccal mucosa grafts (BMGs) are hairless, thin, and easy to harvest; they re-establish the dry–wet border with high take on vascular beds and minimal commissure tethering. In a 16-patient series pairing mental V-Y skin reconstruction with mucosal options, BMGs yielded good esthetic and functional outcomes in all BMG cases, whereas mucosal V-Y flaps occasionally caused oral incompetence, and buccal mucosal flaps produced commissure deformity [29]. Labial or buccal mucosal advancement flaps provide excellent texture and pliability for partial-thickness or hemivermilion defects, though over-advancement may narrow the oral aperture. For larger spans or compromised beds, the facial artery musculomucosal (FAMM; buccinator-based) flap supplies vascularized mucosa on a reliable pedicle. It has broad application to lip reconstruction—including pediatric cases—while requiring attention to Stensen’s duct and the cheek bulk [30,31]. When mucosal reconstruction is insufficient or contraindicated, cutaneous or myocutaneous flaps (e.g., V-Y [32], Estlander [33], Abbe [24], Karapandžić or Colmenero [34]) address adjacent cutaneous deficits. These techniques often yield exceptional color, texture, integration with the native lip, and border control but carry trade-offs such as microstomia, commissure malposition, or sphincter imbalance, depending on defect geometry. A nasolabial flap offers reliable vascularity and generous reach for cutaneous lower-lip resurfacing near the commissure, with potential drawbacks of hair-bearing skin in males and donor-site scarring. A buccinator (myo)mucosal flap provides pliable intraoral tissue with good mobility for perioral reconstruction [35]. Free tissue transfer (e.g., prefabricated gracilis muscle free flap [36]) is reserved for composite, extensive, high-morbidity defects [37]. Novel biologic materials (e.g., cryopreserved umbilical cord allografts) remain investigational [38].

Debridement plays a crucial role in preserving tissue vitality, which was performed surgically in this case; however, alternatives, e.g., low-frequency contact ultrasound (US) or enzymatic necrolysis (bromelain), may result in enhanced biofilm penetration [39]. Prophylactic antibiotics were used in this case to mitigate the high risk of wound infection associated with mammalian bites, a strategy supported by the existing literature. Nonetheless, recent analyses suggest potential over-prescription in bite injuries; protocolized stewardship is warranted [8].

In our procedure, PHMB was used both as an irrigant during debridement and as a gel beneath a PU foam dressing. Based on in vitro data, above 10 micrograms per milliliter (µg/mL), PHMB has broad-spectrum bactericidal effects, while lower doses are only bacteriostatic [40]. Though PHMB enhances keratinocyte proliferation and displays low mammalian cell toxicity at ≤2 µg/mL, its biphasic dose–response curve reveals a critical limitation. Concentrations exceeding that threshold induce dose-dependent suppression of cell multiplication, potentially impairing re-epithelialization in heavily exuding wounds. Therefore, we applied a higher initial concentration to disrupt suspected biofilm, followed by a lower-dose maintenance phase; however, tissue levels were not measured, so mechanistic effects cannot be confirmed.

A PU dressing was chosen due to its even, durable compression capabilities over a broad, curved surface and its availability. No negative pressure was applied; the foam served solely for external compression and immobilization, resulting in adequate graft take. Additionally, no dressing changes were necessary during the first postoperative week, minimizing pain and discomfort.

Long-term outcomes for pediatric lower lip dog bite injuries are generally positive, with most patients regaining function despite persistent scarring and emotional impacts that require ongoing care [22,41]. Advances in pediatric plastic surgery and refined scar management continue to have improved results, though psychological recovery often lags behind physical healing, highlighting the need for early psychosocial intervention [25]. This case also underscores the public health importance of prevention measures such as responsible pet ownership, vaccination, and education on safe animal interactions. Ultimately, a multidisciplinary approach combining surgical expertise, infection control, and long-term follow-up is essential for optimal outcomes.

Several limitations of this case report must be acknowledged. First, this is a single-patient report without a comparator, which limits external validity and precludes causal inference. Follow-up to 12 months captures early–mid-term healing but is insufficient to assess growth-related changes, graft contraction, or late esthetic and functional outcomes. Outcomes were based on clinical examination and serial photographs only. We did not record standardized endpoints (e.g., Vancouver Scar Scale (VSS), Patient and Observer Scar Assessment Scale (POSAS), Pediatric Quality-of-Life Inventory (PedsQL), pain levels, lip strength, drooling, or water-holding tests). No objective perfusion/biomechanical tests (e.g., Doppler US, elastometry) nor digital colorimetry were performed. Mechanistic data were not collected (e.g., tissue PHMB concentrations, edema metrics). The favorable course reflects the combined intervention; therefore, the contribution of individual components cannot be determined. Outcomes may vary depending on defect geometry and location, or patient age, gender, and compliance. Imaging and intra-operative documentation were limited in angle and detail. Finally, although FTSG was acceptable here, mucosal and cutaneous flap options, non-surgical debridement, and other antimicrobial or dressing strategies may offer superior results. However, these alternatives were not evaluated due to the rarity of this condition.

## 5. Conclusions

In this single descriptive case of an extensive pediatric lower-lip dog bite injury, primary closure—using sharp debridement, systemic antibiotics, FTSG with a suture-anchored hydrophobic PU compression foam bolster, and adjunct PHMB—was associated with an uncomplicated functional course and durable coverage at 12 months. Mild esthetic sequelae persisted (shallow indentation, vellus hair within the graft, partial vermilion-border effacement with mild dyschromia). Consequently, elective secondary refinement may be considered.

Because multiple interventions were applied concurrently and no validated outcome scales were used, the contribution of individual components cannot be determined. Prospective, randomized, comparative studies using standardized functional, esthetic, and patient-reported outcomes, with objective assessments (e.g., perfusion), are needed.

## Data Availability

Data are contained within the article.

## Abbreviations

The following abbreviations are used in this manuscript:

BMGs	Buccal mucosa grafts
FAMM	Facial artery musculomucosal (flap)
FTSG	Full-thickness skin grafting
µg/ml	micrograms per milliliter
mm	millimeter
NPWT	Negative-Pressure Wound Therapy
PedsQL	Pediatric Quality-of-Life Inventory
PHMB	Polyhexamethylene biguanide (polyhexanide)
POSAS	Patient and Observer Scar Assessment Scale
PT	Physical therapy
PU	Polyurethane
TIVA	Total intravenous anesthesia
US	Ultrasound
VSS	Vancouver Scar Scale
